# In *Rhodotorula mucilaginosa*, active oxidative metabolism increases carotenoids to inactivate excess reactive oxygen species

**DOI:** 10.3389/ffunb.2024.1378590

**Published:** 2024-09-06

**Authors:** Edson Mosqueda-Martínez, Natalia Chiquete-Félix, Paulina Castañeda-Tamez, Carolina Ricardez-García, Manuel Gutiérrez-Aguilar, Salvador Uribe-Carvajal, Ofelia Mendez-Romero

**Affiliations:** ^1^ Department of Genetics and Molecular Biology, Instituto de Fisiología Celular, Universidad Nacional Autónoma de México, Mexico City, Mexico; ^2^ Department of Biochemistry, Facultad de Química, Universidad Nacional Autonoma de México, Mexico City, Mexico

**Keywords:** carotenoids, *Rhodotorula mucilaginosa*, carbon source, aerobic metabolism, diphenylamine (DPA), ROS

## Abstract

Carotenoids produced by bacteria, yeasts, algae and plants inactivate Free Radicals (FR). However, FR may inactivate carotenoids and even turn them into free radicals. Oxidative metabolism is a source of the highly motile Reactive Oxygen Species (ROS). To evaluate carotenoid interactions with ROS, the yeast *Rhodotorula mucilaginosa* was grown in dextrose (YPD), a fermentative substrate where low rates of oxygen consumption and low carotenoid expression were observed, or in lactate (YPLac), a mitochondrial oxidative-phosphorylation (OxPhos) substrate, which supports high respiratory activity and carotenoid production. ROS were high in YPLac-grown cells and these were unmasked by the carotenoid production-inhibitor diphenylamine (DPA). In contrast, in YPD-grown cells ROS were almost absent. It is proposed that YPLac cells are under oxidative stress. In addition, YPLac-grown cells were more sensitive than YPD-grown cells to menadione (MD), a FR-releasing agent. To test whether carotenoids from cells grown in YPLac had been modified by ROS, carotenoids from each, YPD- and YPLac-grown cells were isolated and added back to cells, evaluating protection from MD. Remarkably, carotenoids extracted from cells grown in YPLac medium inhibited growth, while in contrast extracts from YPD-grown cells were innocuous or mildly protective. Results suggest that carotenoid-synthesis in YPLac-cells is a response to OxPhos-produced ROS. However, upon reacting with FR, carotenoids themselves may be inactivated or even become prooxidant themselves.

## 1 Introduction

Life is found almost anywhere on Earth (Merino et al., 2019). Organisms have developed systems to survive in extreme conditions such as high and low proton and salt concentrations or extreme temperatures (Coleine et al., 2022; Touchette et al., 2022; Liu et al., 2023; Yoo et al., 2023). Oxygen is an ideal electron acceptor that releases large amounts of energy during its catalyzed reduction (Mendez-Romero et al., 2022). However, its non-catalyzed partial reduction produces highly reactive Free Radicals (FR) known as Reactive Oxygen Species (ROS) (Sies et al., 2022). ROS damage proteins, lipids and nucleic acids, leading to cell dysfunction and eventual death (Li et al., 2018). Cells have developed multiple mechanisms to prevent ROS toxicity: some, such as physiological uncoupling, prevent ROS generation (Guerrero-Castillo et al., 2011; Cabrera-Orefice et al., 2014; Castañeda-Tamez et al., 2024). Other systems deactivate ROS enzymatically: these are superoxide dismutase, catalases and glutathione reductase (Jamova et al., 2024). A third class of ROS detoxification system includes pigments like chlorophylls, melanin and carotenoids, that react with FR to inactivate them (Priyadarshini Pradhan et al., 2022; Tamiaki, 2022; Suthar et al., 2023). These pigments quench and inactivate ROS, protecting proteins, membranes, and DNA (Stahl and Sies, 2003; Salman et al., 2007; Choi and Lee, 2015; Lucas et al., 2020). In humans, ingested carotenoids protect against cancer and illnesses that include cardiovascular disorders, cataracts, age-related macular degeneration, osteoporosis, and diabetes (Milani et al., 2017; Shabhir and Nuzhat, 2018; Paul et al., 2023).

*Rhodotorula* spp. fungi (Moliné et al., 2010; Irazusta et al., 2013; Chen et al., 2022) thrive in diverse harsh environments, including soils, contaminated waters, and permafrost layers (Ge et al., 2021). When exposed to UV-radiation, hyperosmolarity or ROS, these species enhance carotenoid synthesis (Bhosale and Gadre, 2002; Aksu and Eren, 2005; Garcia-Cortes et al., 2021; Li et al., 2022). Under these conditions, the DPA-mediated inhibition of carotenoid production decreases survival (Moore et al., 1989; Moliné et al., 2010). It has been suggested that pigmented yeasts of the genera *Rhodotorula*, *Sporobolomyces*, *Phaffya* and *Cystofilobasidium* synthesize carotenoids to compensate for deficiencies in other antioxidant systems, such as copper and zinc superoxide-dismutase (Cu/Zn-SOD) (Moore et al., 1989; Schroeder and Johnson, 1993; Moliné et al., 2009). While *R. mucilaginosa* does possess the gene for Cu/Zn-SOD, it does not express it under basal conditions (Hernández-Saavedra, 2003).

Carotenoids may be non-substituted hydrocarbons such as β-carotene and torulene, or xanthophylls, oxygenated derivatives like thorularhodin (Watcharawipas and Runguphan, 2022; Paul et al., 2023). Carotenoids inactivate ROS through two possible mechanisms: the first one involves dissipating energy into the surrounding medium as heat, returning singlet oxygen (^1^O_2_) to its basal state without altering the carotenoid (Stahl and Sies, 2003). The second mechanism involves electron transfer, where carotenoids are oxidized and inactivated; these oxidized species cannot be recycled (Ribeiro et al., 2018). Carotenoid reactions can be hazardous as they may produce pro-oxidizing derivatives that damage cell structures (Henry et al., 2000; Lucas et al., 2020). Inhibitors of carotenoid biosynthesis, such as diphenylamine (DPA), block the sequential desaturation of phytoene (Clarke et al., 1983; Moliné et al., 2012) and are used to assess the role of carotenoids in the cell (Maxwell et al., 1966; Hayman et al., 1974).

We added different DPA concentrations to *R. mucilaginosa* to evaluate carotenoid protection against ROS. Cells grown in lactate as the carbon source produced more carotenoids than those using dextrose. Dextrose is a fermentative substrate that requires little mitochondrial activity (Castañeda-Tamez et al., 2024). YPLac-grown cells exhibited higher oxygen consumption rates and were under oxidative stress, as indicated by increased carotenoid synthesis. DPA was added to inhibit carotenoid production, unmasking ROS concentrations. Higher ROS were found in YPLac-grown cells. Additionally, YPLac-grown cells were more sensitive to menadione. When added back to new cells, isolated carotenoids from YPD-grown cells exhibited a mild protective effect, while those from YPLac-grown cells inhibited growth partially. These findings suggest that, increased carotenoid synthesis constitutes a response to oxidative stress in *R. mucilaginosa*. However, during ROS deactivation, some carotenoids are probably modified, losing their protective activity and even producing pro-oxidizing species (Lucas et al., 2020).

## 2 Materials and methods

### 2.1 Yeast and culture media

All reagents were analytical grade. *Rhodotorula mucilaginosa* ATCC 66034 was kept at room temperature in Petri dishes containing YPD agar (10 g yeast extract, (MCD Lab, Estado de México, Mexico) 20 g peptone (MCD Lab, Estado de México, Mexico), 20 g glucose (Sigma Chem Co, St. Louis Mo, USA) and 20 g agar (Difco, Detroit Mi, USA). Cells were used within three weeks. For experiments, a loophole was inoculated into 10 mL of YPD (1% yeast extract, 2% peptone, 2% dextrose) or YPLac (1% yeast extract, 2% peptone, 2% lactate, pH 6.0. For YPLac, titration of pH to 6.0 with NaOH was needed to neutralize added 85% lactic acid (Meyer, CDMX, Mexico) and grown overnight. Then, an aliquot was added to 100 mL of the corresponding medium to an O.D. = 0.05 (540 nm). Note that the final concentration for each carbon source was 2%, *i.e*., 0.11 M dextrose or 0.23 M lactic. Flasks were incubated in a Gyratory Shaker (G10 model, New Brunswick Scientific, New Jersey, USA) at 250 rpm and 30°C for 24 hours. All experiments were performed in triplicate. Carotenoid production was inhibited by adding different concentrations of diphenylamine (DPA) (Sigma-Aldrich, Darmstadt, Germany) as described by Moliné et al. (2010). To discard any vehicle effects, we adjusted DPA concentrations in stock solutions (e.g., a 3.75 mM DPA solution was used to add 4 μL/mL and attain 15 μM DPA). We always added 4 µl EtOH/mL alone in controls to discard any effects on growth or oxygen consumption (See below).

### 2.2 Growth curves

Cells were seeded introducing a loophole from a Petri dish culture into 50 mL of either YPD (where dextrose is a fermentable carbon source) or YPLac (where lactate is a non-fermentable carbon source) (Castañeda-Tamez et al., 2024). After 24 h, cells were added to 100 mL of the corresponding medium, adjusting concentration to O.D. = 0.05 and were cultivated at 30°C. We used 250 mL Erlenmeyer flasks modified in our glass shop by attaching a Klett-test tube to the wall (Pinocchios) and cell growth was evaluated every three hours in a Klett-Summerson Model 800 colorimeter (Green filter) (Klett Manufacturing Co., New York, USA). To discard any effects of DPA on growth, samples containing 15 and 40 µM DPA were also tested (**Supplementary Figure S1**). In addition to absorbance data, biomass wet weight was measured. Although, dry weight is probably more accurate, wet weight measurement is very straightforward and is routinely used to produce an estimate of cell mass (Uribe et al., 1985; Godbey, 2022). Cells were harvested and washed with distilled water three times at 6000 *x g* for 5 min at 4°C and then, samples were centrifuged at 12,000 *x g* for 5 min and the supernatant was discarded (Mussagy et al., 2021a). Subsequently, pellets were weighed using a Highland^®^ Portable Precision Balance-HCB 602H (ADAM, Oxford, USA).

### 2.3 Rate of oxygen consumption

To test oxidative metabolism, the rate of oxygen consumption was measured in cells harvested at 24 h (Log phase) grown in either YPD or YPLac (Purvis and Gegogeine, 2003). Respiration buffer was 10 mM 4-morpholineethanesulfonic acid (MES) pH 6.0. Cells were added to a final concentration of 12.5 mg (ww)/mL (Dejean et al., 2000). Additions were: one minute after initiating a given trace 40 µM DPA and after another minute 32 µM carbonyl cyanide 3-chlorophenylhydrazone (CCCP) (Barrientos, 2002) (see **Supplementary Figure S2**). Measurements were made using a Clark-type electrode coupled to an oximeter (StrathKelvin instruments model 782, North Lanarkshire, Scotland) equipped with a 1 mL water-jacketed chamber. Temperature was kept at 30°C with a water bath (PolyScience 7, Warrington Pa, USA). Oxygen uptake was measured as a function of time from the tangent to the initial part of the progress curve and expressed as nanoatom-grams of oxygen per minute per milligram of cells (wet weight) (natgO min^-1^. mg cells (ww)^-1^) (Bari et al., 2010; Nicholls and Ferguson, 2013).

### 2.4 Carotenoid extraction and quantitation

Under stress, *R. mucilaginosa* increases carotenoid production. To evaluate this, carotenoids were extracted from 24 h cells using a microwave method as described by Mohamadi et al. (2013) with slight modifications. Briefly, cells were washed with distilled water three times at 6000 *x g* for 5 min at 4°C. Then, samples were centrifuged at 12000 *xg* for 5 min and the supernatant was discarded while the pellet (1 g (ww) mL^-1^) was spread on the surface of a glass Petri dish. Each dish was treated in a microwave oven with a concave reflection system for 30 sec at 700 watts (Daewoo, Seoul, Korea). A fine pink powder was obtained and dissolved in DMSO to 50 mg dry weight per mL. The sample was sonicated for 30 min (Sonics Vibra Cell, Newtown, CT, USA) at 20 kHz, 50% amplitude with pulses of 30 sec alternated with 30 sec resting periods on ice. These were incubated under agitation for 1 h at room temperature and then cyclohexane, 5 mL/0.1 g dry weight biomass was added and further incubated for 60 min at room temp. Extraction was performed twice. At the end, the sample was centrifuged at 12000 *x g* for 10 min and the remaining organic phase was evaporated under a mild airflow (3 L/min) in a dark chamber until a dry powder was obtained. Each sample was solubilized in 0.2 mL 96% ethanol (Jaeschke et al., 2017) and absorbance spectra, from 400 to 600 nm (POLARstar Omega luminometer, BGM LABTECH, Allmendgrün, Germany) were taken. Carotenoid identities were annotated as in Varmira et al. (2016). To avoid interference with the torularhodin peak at 480 nm, torulene was identified by its characteristic shoulder at 530 nm instead of 490 nm, Carotene concentration was determined as in Sharma and Ghoshal (2020).

### 2.5 Thin layer chromatography

TLC was used to estimate of carotenoid composition on YPD- and YPLac-cell extracts. The stationary phase was silica gel in commercial plates (TLC silica gel 60 F254, 6x9 cm (Merck, Darmstadt, Germany). The mobile phase was ether:hexane:acetone (90:30:10, v/v/v; Meyer, CDMX, Mexico). Samples were run for 20 min at room temperature (Kanno et al., 2021). Images were taken in visible light. The distance (Rf) between the baseline and each spot was estimated, and spots were tentatively identified comparing with the literature (Zeb and Murkovic, 2010; Cheng and Yang, 2016). In an effort to further explore carotenoid identity, each band from TLC was excised and eluted in the same solvent and its absorbance spectrum was read at 400 to 600 nm in a POLARstar Omega luminometer. Detected pigments were annotated as in Moliné et al. (2012).

### 2.6 Survival under oxidative stress

*R. mucilaginosa* survival was evaluated in a dilution spot assay of cells grown in either YPD or YPLac at 1.0 O.D. First, cells were grown in 100 mL, at 250 rpm at 30°C in the presence of 0, 15 or 40 µM DPA (Sigma, USA) (Jamieson, 1992). At 15 μM DPA carotenoid production was inhibited by 80%, while as 40 mM DPA it was inhibited by 100%. After 24 hours, 0, 15 or 40 mM menadione (MD), a free-radical producing agent was added (Sigma-Aldrich, Darmstadt, Germany) and the mixture was further incubated under agitation for 2 more hours in an orbital shaker at 250 rpm (G10, New Brunswick Sci, NJ, USA). Then, samples were collected and concentration adjusted to O.D. = 1.0. These cells were used to conduct a spot assay using a 96 well plate with 200 μL in each well. Then, performing 1/10 serial dilutions in the same medium where they grew (either YPD or YPLac) (dots, from left to right in each panel). All samples were incubated at 30°C for three days. Petri dishes were distributed as follows: Cells grown in either YPD (upper panels) or YPLac (lower panels) were divided into three groups: No additions, Medium supplemented with 15 µM DPA, and Medium supplemented with 40 µM DPA. In each panel, rows were as follows: row 1, no additions; row 2, DMSO alone; row 3, 15 mM MD and row 4, 40 mM MD.

### 2.7 Reactive oxygen species quantitation

ROS concentrations at different carotenoid concentrations were measured in cells grown in the presence of different DPA concentrations (0 to 40 µM) (Moore et al., 1989; Irazusta et al., 2013; Tang et al., 2019). In each sample, both, carotenoids and ROS were measured. The reaction buffer (0.25 M Na_3_PO_4_, pH 7.4) was complemented with 10 μM Amplex^®^ Red, hydrogen peroxide/peroxidase kit (Invitrogen, Waltham Ma, USA), 0.2 U horseradish peroxidase/mL and 0.2 U superoxide dismutase/mL (Zhou et al., 1997). Cells from each medium were harvested and washed with distilled water three times at 6000 *xg* for 5 minutes at 4°C and then these were aliquoted in 5 mM 4-(2-hydroxyethyl)-1-piperazine-ethanesulfonic acid (HEPES) pH 7.0 in a small Eppendorf tube (1.5 mL), mixed 50/50 v/v with 0.5 mm glass beads, vortexed for 3 min and solubilized with sodium deoxycholate. Protein concentration in homogenates was measured by biuret (Gornall et al., 1949). From each suspension, 100 µg protein/well was added to a POLARstar Omega luminometer (BGM LABTECH) and samples were incubated for 40 min and read against a H_2_O_2_ standard curve (0 to 200 nmol) made in 5 mM HEPES pH 7.0 (Guerrero-Castillo et al., 2011; Morales-García et al., 2021). Experiments were conducted in triplicate and data are reported as H_2_O_2_ nmol/µg protein ± SD. See 2.9 for statistical analysis. Carotenoids were measured as described above (2.4).

### 2.8 Carotenoid extract prooxidant effect

To evaluate if carotenoids preserved protective activity after exposure to stress, these pigments were recovered from either YPLac- or YPD-grown cells (see section 2.4) sealed under a N flow and stored in the dark at -20°C. Cells grown in either YPD or YPLac plus 15 µM DPA produced a small amount of endogenous carotenoids (20% as compared to the control). After 24 h, 40 mM menadione without or with 40 µg/g (dry weight) cell carotenoid extract (from either YPD or YLac-cultures) were added to the new cells further incubating for 2 hours at 30°C. After incubation, cells were used in a Colony Forming Unit (CFU) assay (Tran and Green, 2019; Suarez-Diez et al., 2020). Briefly, 50 µL of a 10^-5^ cell dilution were added to YPD or YPLac agar plates and incubated for 3 days at 30°C. Then, CFUs were counted. Results are reported as percentage of CFUs against a control without added carotenoids and menadione (Bhuyan et al., 2023).

### 2.9 Statistical analysis

Statistical differences were evaluated using one-way ANOVA (Fisher, 1992). Significant differences between means were evaluated with Fischer’s multiple comparison test to *p<0.05*. Data analysis and graphics were constructed with GraphPad Prism for Windows, version 8.0.2 (263).

## 3 Results

### 3.1 *Rhodotorula mucilaginosa* grew more in dextrose than in lactate

**Figure 1:**
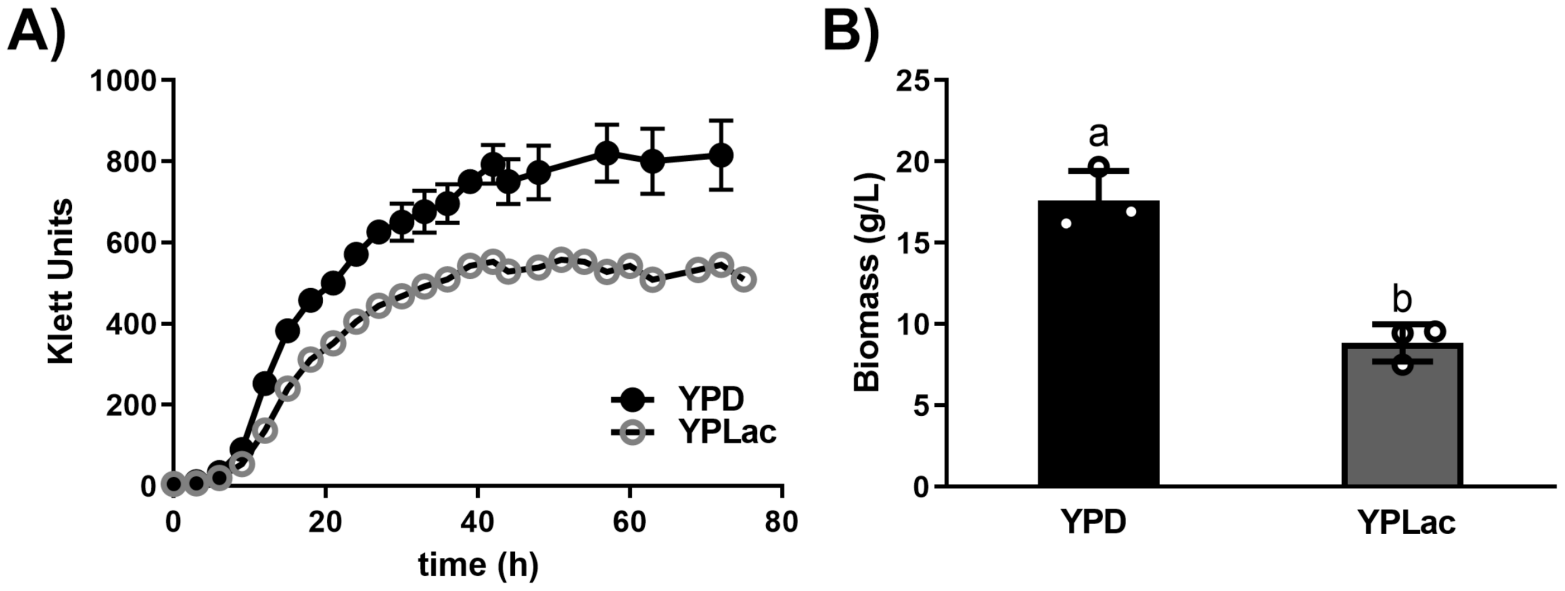
*Rhodotorula mucilaginosa* growth curves and biomass. **(A)** Growth of *R. mucilaginosa* was evaluated using a Klett-Summerson colorimeter (Green filter) in the presence of either a fermentable carbon source YPD (black dots) or a non-fermentable carbon source YPLac (gray circles). When SD bars were smaller than the illustrated dots, they were omitted. **(B)** Biomass of samples taken at 24 h (wet weight). Data are mean ± SD (n= 3): different letters indicate a significant difference (p<0.05).

Growth curves for *R. mucilaginosa* (**Figure 1A**) were complemented by biomass measurements in cells cultured for 24 h (**Figure 1B**). YPD-grown cells reached 570 Klett units at 24 h (mid-Log phase) while YPLac-grown cells reached 405 Klett units. The stationary phase was reached in both cases around 40 h, reaching 810 Klett units for YPD-grown cells and 542 Klett units for YPlac-cells (**Figure 1A**). YPD-cell biomass at 24 h was 17.6 g (ww)/L while YPLac-cells weighed 8.8 g (ww)/L (**Figure 1B**). Thus, cells grown in YPD grew about 1.8 times as much as YPLac-grown cells. This is comparable to reports by others (Elsanhoty et al., 2017) and to results from other yeasts such as *S. cerevisiae* (De Barros et al., 2023). All experiments were conducted in cells grown until mid-Log phase (24 hours). As DPA was used in other experiments, its effect on growth was tested. Consistent with findings in the literature (Moore et al., 1989; Irazusta et al., 2013), DPA did not affect growth in *R. mucilaginosa* (**Supplementary Figure S1**).

### 3.2 The rate of oxygen consumption was higher in YPLac- than in YPD-grown cells

**Figure 2:**
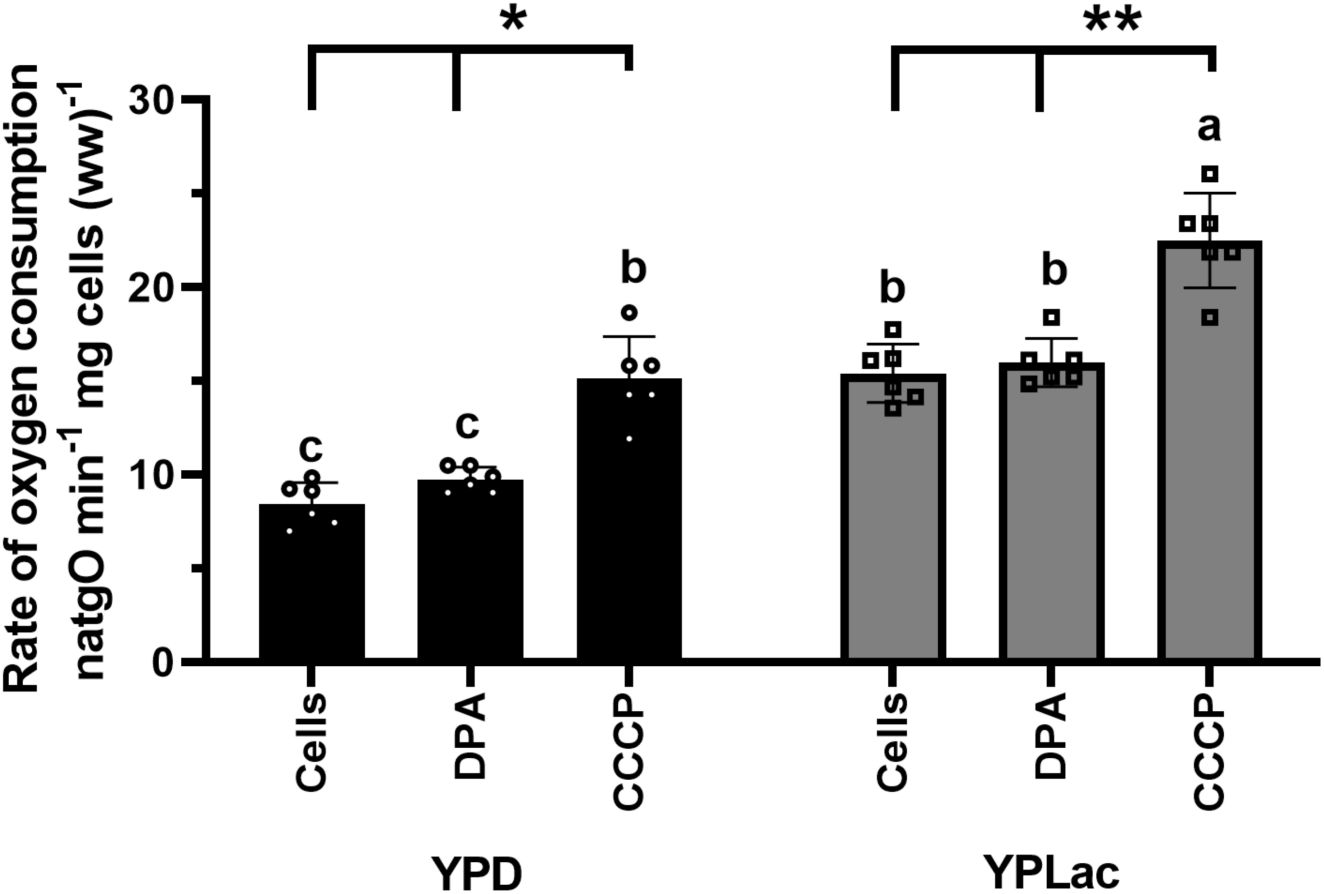
Rate of oxygen consumption by *R. mucilaginosa* cells. Cells grown in either YPD- or YPLac-media for 24 h. were used. Reaction mixture: 10 mM 4-morpholineethanesulfonic acid (MES), pH 6.0. 2% dextrose. Where indicated, 40 μM DPA or 32 μM CCCP. Cells 12.5 mg ww/mL Data are means ± SD (n=6). Statistical differences (one-way ANOVA) between YPD-cells and YPLac-cells are indicated with Latin letters. Asterisks indicate differences within a specific medium comparing the uncoupled state (CCCP) against their respective basal respiratory activity. In all cases, *p*<0.0001.

Oxygen consumption was measured in YPD- and in YPLac-cells both in basal conditions and at a maximal flow of electrons evoked by the OxPhos-uncoupler CCCP (**Figure 2**; **Supplementary Figure S2**). DPA was also tested, and it did not have any effects (**Figure 2**; **Supplementary Figure S2**). The basal rate of oxygen consumption for YPD-grown cells was 8 natgO min^-1^. mg cells (ww)^-1^ and 15 natgO min^-1^. mg cells (ww)^-1^ in the uncoupled state (**Figure 2**, black bars). In YPLac-grown cells the basal rate of oxygen consumption was 15 natgO min^-1^. mg cells (ww)^-1^ and when CCCP was added it increased to 23 natgO min^-1^. mg cells (ww)^-1^ (**Figure 2**). Thus, both in YPD-grown cells (**Figure 2**, black bars) and YPLac-grown cells (**Figure 2**, gray bars), the uncoupled rate of respiration was higher than in the basal state, while DPA had no effects. In addition, in all cases the rate of oxygen consumption was higher in YPLac- than in YPD-grown cells (**Figure 2**), indicating cells were well coupled. Recently, it was reported that in *R. mucilaginosa* the mitochondrial respiratory chain components vary in concentration, depending on whether the growth medium is YPLac or YPD (Castañeda-Tamez et al., 2024). The higher rate of oxygen consumption observed in YPLac-grown cells suggested that ROS increased.

### 3.3 Carotenoid concentration was higher in YPLac- than in YPD-grown cells

**Figure 3:**
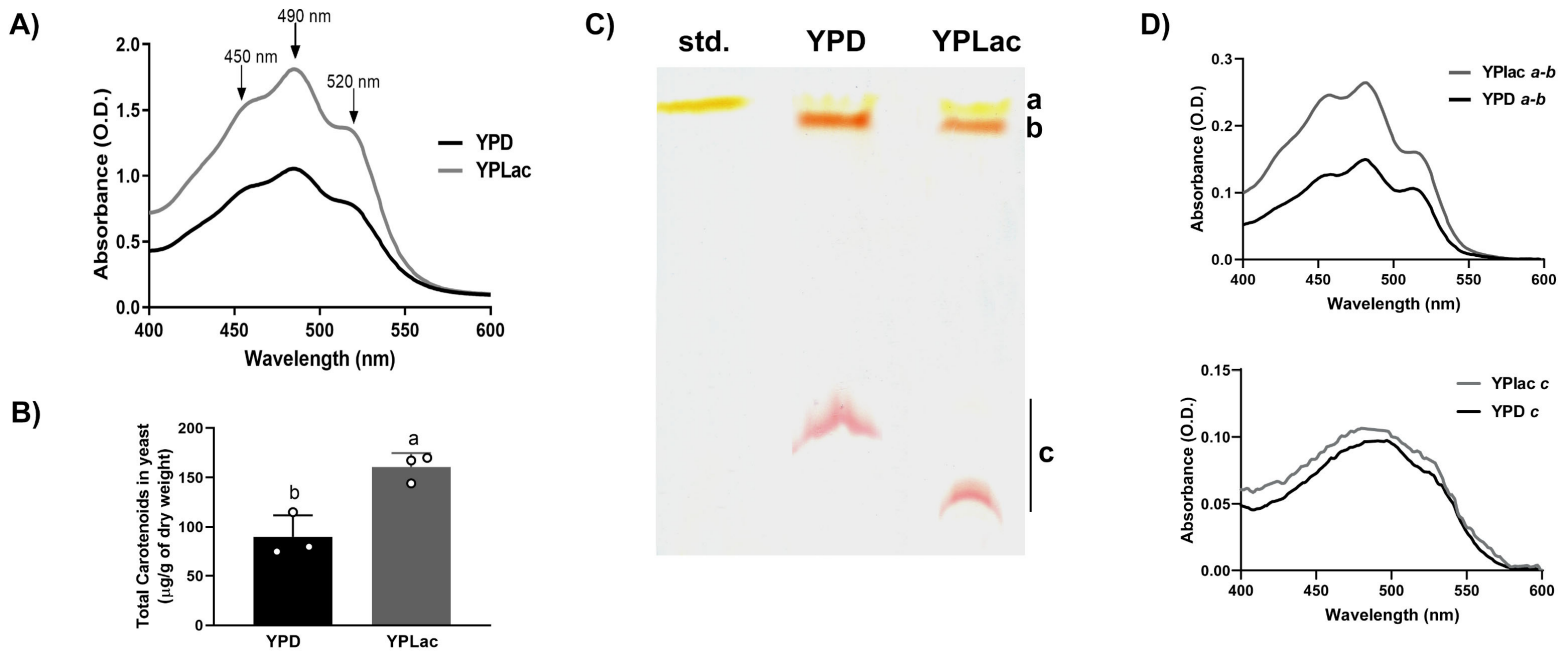
Carotenoid production by *R. mucilaginosa* cells. **(A)** Absorbance spectra of carotenoids extracted from *R. mucilaginosa* grown in YPD (black line) or YPLac (gray line) at the Log phase. Peaks corresponding to carotenoids typically obtained from *R. mucilaginosa* are indicated with arrows: β-carotene (450 nm), Torularhodin (490 nm) and Torulene (520nm). **(B)** Total carotenoid estimated from spectra taken under each condition, as indicated; data are mean ± SD (n=3). Different letters indicate a significant difference (*p<0.05*). Where: dextrose (black) and lactate (gray). **(C)** TLC of carotenoid extracts from YPD- or YPLac-grown *R. mucilaginosa* cells. For comparison, a commercial sample of β-carotene was also included. Different colored spots were observed and labeled as follows: a, Rf = 0.92; b, Rf = 0.85; and c Rf= 0.33 (YPD) and 0.14 (YPLac), respectively. The mobile phase used was petroleum ether:hexane:acetone, in a ratio of 90:30:10 v/v. **(D)** Absorbance spectra from TLC spots *a + b* from either YPD- and YPLac-extracts and of spot *c* from either YPD- and YPLac -extracts. In all cases, YPD-extract traces are in black, while YPLac-extract traces are in gray). Data are representative (n= 3).

*R. mucilaginosa* cells grown in YPLac medium were orange, while YPD-grown colonies were pale pink (See **Figure 3**, “No addition” rows) suggesting that carotenoid production was higher in cells grown in YPLac medium. Thus, we decided to evaluate carotenoid concentrations by extracting them from either YPD- (**Figure 3A**, black trace) or YPLac-grown cells (**Figure 3A**, gray trace) and running absorbance spectra from 400 to 600 nm (Mussagy et al., 2021b). Carotenoids extracted from YPD-grown cells presented an initial absorbance nearing 0.5 units at 400 nm, and an absorbance increase reaching a peak at 490 nm. At higher wavelengths, absorbance decreased except for a shoulder at 520 nm, nearing zero at 600 nm. Under YPLac growth conditions, initial absorbance was close to 0.75 units and steadily increased until maxing out at 490 nm and decreasing at wavelengths higher that 520 nm. Notably, spectra exhibited peaks that were like those reported for *Rhodotorula sp*, *i*.*e*., β-carotene (λmax 450 nm), torularhodin (λmax 490 nm), and torulene (λmax 520 nm) (Park et al., 2007; Varmira et al., 2016; Udensi et al., 2022). To have a rough estimate of carotenoid concentrations in these samples, the extinction coefficient 0.16 cm^-1^ M^-1^ was used as in Sharma and Ghoshal, 2020 and Mussagy et al., 2021a (**Figure 3B**). In YPD samples, carotenoids were 90 µg/g cells dry weight, and in YPLac-grown samples 161 µg/g cells dry weight. The large increase in carotenoid synthesis observed in YPLac-grown cells suggested that these were under high oxidative stress.

To further characterize carotenoid production in *R. mucilaginosa*, a TLC assay was performed (**Figure 3C**). Extracts from either YDP- or YPLac-grown cells were included, along with a β-carotene standard (Std.). Both extracts revealed three colored bands, with bands *a* and *b* running very close to each other and to the large band in the standard (Rf= 0.92 to 0.85). A third band (*c*) with decreased migration was also detected in YPD- (Rf= 0.33) and in YPLac-growth extracts (Rf=0.14). All bands were scrapped from the silica plate, and their absorbance spectra were analyzed from each, YPD- (**Figure 3D**, black traces) or YPLac (**Figure 3D**, gray traces). Bands *a* and *b* were too close, so they were pooled together. The spectra revealed two peaks at 450 and 484 nm, suggesting the presence of a mixture of β-carotene (450 nm) and torulene (484 nm) (**Figures 3C, D**, top panel) (Moliné et al., 2012). In YPLac-cells absorbance was higher than in YPD-cells. In addition, an absorbance shoulder at 520 nm was proportionally decreased only in YPLac, suggesting that carotenoid contents were different (**Figure 3D** traces for bands *a* and *b*). The lower band *c* ran at slightly different Rfs depending on whether it came from YPD- or YPLac-grown cells. However, the deep red color and the curved shape of both bands suggested that it was the same carotenoid. This was tested running separate spectra for bands *c* from each, YPD- (**Figure 3D** bottom spectrum, black trace) or YPLac (**Figure 3D** bottom spectrum, gray trace). Spectra from bands *c* were almost superimposable. In addition, these exhibited a peak at 490 nm, suggested that both bands *c* were the same pigment, possibly torularhodin. These results, together with data from other authors, suggest that all three carotenoids usually found in *R. mucilaginosa* were present in extracts from both YPD- and YPLac-cells (Perrier et al., 1995; Park et al., 2007; Moliné et al., 2012; Cheng and Yang, 2016; Varmira et al., 2016; Kot et al., 2019; Tang et al., 2019; Lucas et al., 2020). Additionally, data suggest that carotenoid proportions vary with the carbon source as observed in the TLC results and spectra (**Figures 3C, D**). Still, TLC results are only suggestive, and further analyses using mass spectrometry are needed to unequivocally identify each band. It is puzzling that band *c* ran different distances in the YPLac or YPD-cell extracts. It is suggested that as torularhodin contains oxygen, it may be more susceptible to modification by ROS, changing slightly its structure and its affinity for the stationary phase, thus exhibiting a different Rf (Britton, 2008).

### 3.4 YPLac-cells were more sensitive to oxidative stress than YPD-cells

**Figure 4:**
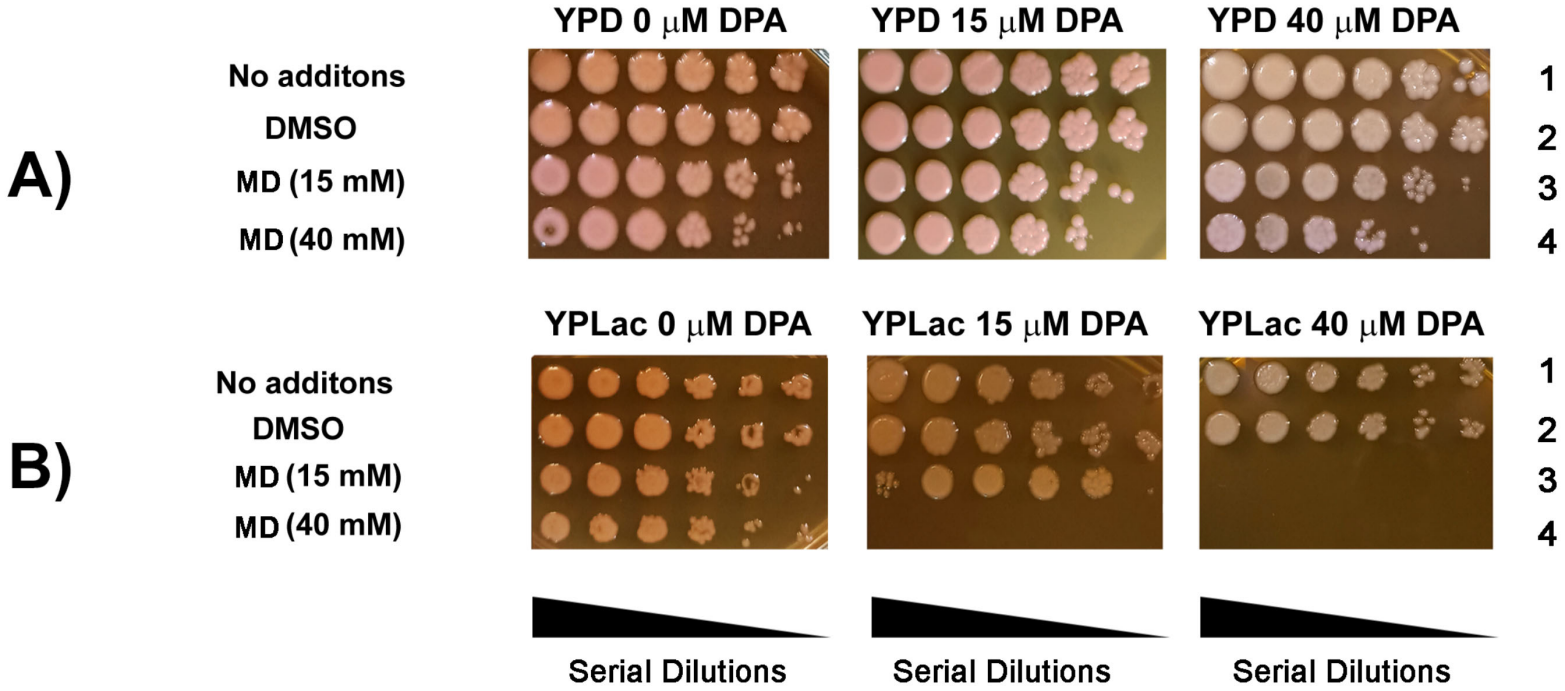
Effect of oxidative stress and inhibition of carotenoid synthesis on *R. mucilaginosa* survival and growth. Spot dilution assay (Serial dilutions: 1, 10^-1^, 10^-2^, 10^-3^, 10^-4^, 10^-5^). Cells were incubated as in **Figure 3** in the presence or absence of DPA. Menadione was added 2 hours before starting the assay. For the assay agar plates were incubated for 24 h at 30°C. **(A)**. YPD-cells; **(B)**, YPLac-cells. Panels: Left No DPA; Center, 15 μM DPA; Right, 40 μM DPA. Rows: Row 1, No additions; Row 2, The vehicle DMSO; Row 3, 15 mM Menadione; Row 4, 40 mM Menadione. Images are representative agar plates (n= 3).

YDP-cell survival was not affected by 15 μM DPA (**Figure 4A**, central panel, row 1) and only mildly by 40 μM DPA (**Figure 4A**, right panel, row 1). In addition, the FR-producing agent MD inhibited growth only slightly (**Figure 4A** rows 3 and 4). In contrast, in YPLac-cells (**Figure 4B**), even in the controls, growth decreased slightly as dilution increased and it was more evident at each DPA concentration (**Figure 4B** all panels, rows 1 and 2). At 40 μM DPA and 15 mM MD, YPLac-cell growth was absent at all dilutions (**Figure 4B**, center panel, row 4) and at 40 μM DPA both MD concentrations fully inhibited growth (**Figure 4B**, right panel, rows 3 and 4). In contrast to YPD-cells, YPLac-cells were highly susceptible to MD, suggesting that they were already under oxidative stress (Biryukova et al., 2009; Tauffenberger et al., 2019). These results suggest that the carotenoid increase in YPLac-cells was due to oxidative stress.

### 3.5 Carotenoid depletion unmasks high ROS production in YPLac-grown cells

**Figure 5:**
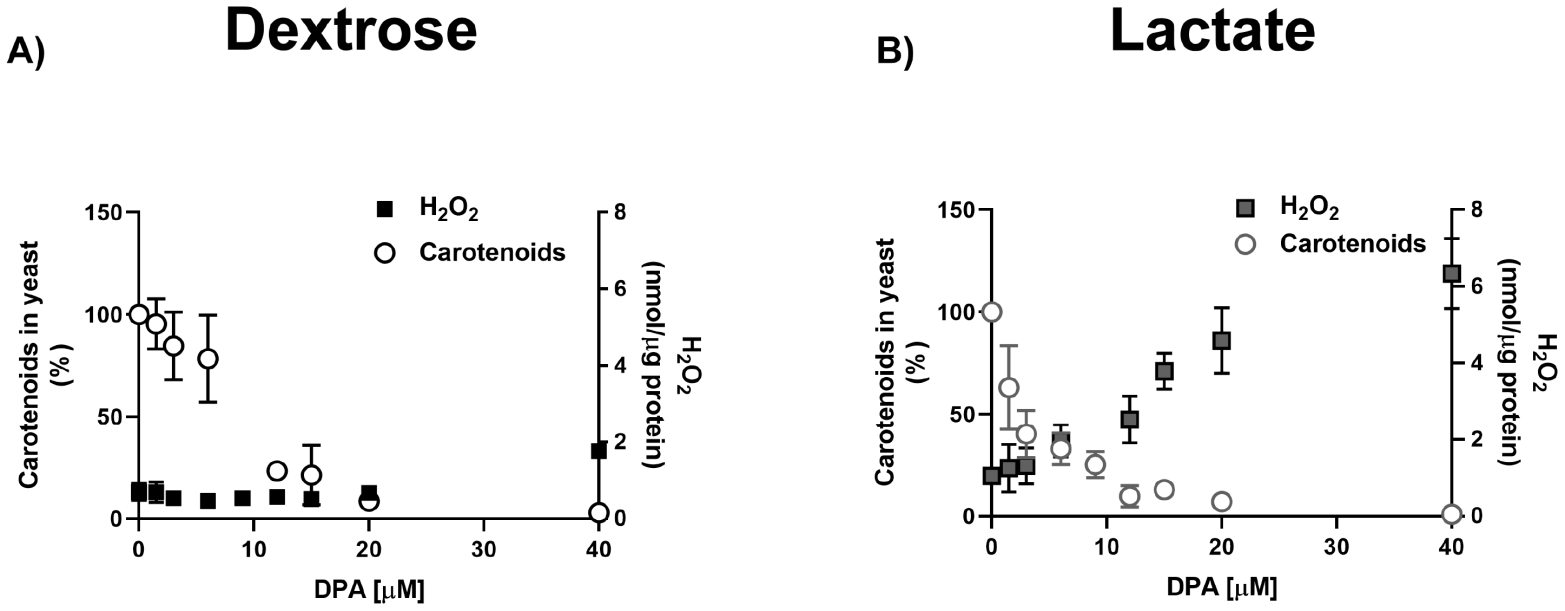
DPA titration of carotenoid and H_2_O_2_ production in *R. mucilaginosa* grown in **(A)** YPD or **(B)** YPLac. Peroxide concentration is expressed as nmol/µg protein and carotenoid absorbance at 490 was estimated. Data are shown as mean ± SD (n=6). Where: carotenoids (dots), peroxide (squares).

In spite of their higher carotenoid content (**Figure 3A**), YPLac-cells were more sensitive to DPA and MD that YPD-cells (**Figure 4**). These results suggest that even control YPLac-cells were under oxidative stress (Castañeda-Tamez et al., 2024). To test this, we decided to unmask ROS production by inhibiting carotenoid synthesis. Thus, we measured both carotenoids and ROS in the presence of 1.5 to 40 μM DPA (**Figure 5**). Carotenoid concentrations are reported as the percentage of absorbance at 490 nm observed in the control, without DPA, which in YPLac-cells was O.D. = 1.75, while in YPD cells was O.D. = 0.95 (See **Figure 3**) (Sharma and Ghoshal, 2020; Mussagy et al., 2021a). At each DPA concentration, measurements of ROS (**Figure 5** black squares) and total carotenoids (**Figure 5**, circles) showed that DPA led to a proportional decrease in carotenoids, both in YPD- (**Figure 5A**) and in YPLac-grown cells (**Figure 5B**). In contrast, ROS concentration variations were different for either YPD or YPLac-cells. In YPD cells, ROS remained below 1.0 nmol H_2_O_2_/µg protein except at 40 µM DPA, a slight increase was observed, to 1.8 nmol H_2_O_2_/µg protein (**Figure 5A**, black squares). In contrast, in YPLac-cells, ROS were already at 1.4 nmol H_2_O_2_/µg protein even without DPA and then, different DPA concentrations led to increased ROS, reaching 6.3 nmol H_2_O_2_/µg protein at 40 µM DPA. Thus, it is proposed that in YPLac-cells carotenoids increased due to high ROS concentrations and DPA unmasked these high concentrations of ROS. In contrast, YPD-grown cells did not exhibit high carotenoid production because they did not produce as much ROS (**Figure 5**).

### 3.6 Carotenoids from YPD- or YPLac-cells showed different effects on fresh cell cultures

**Figure 6:**
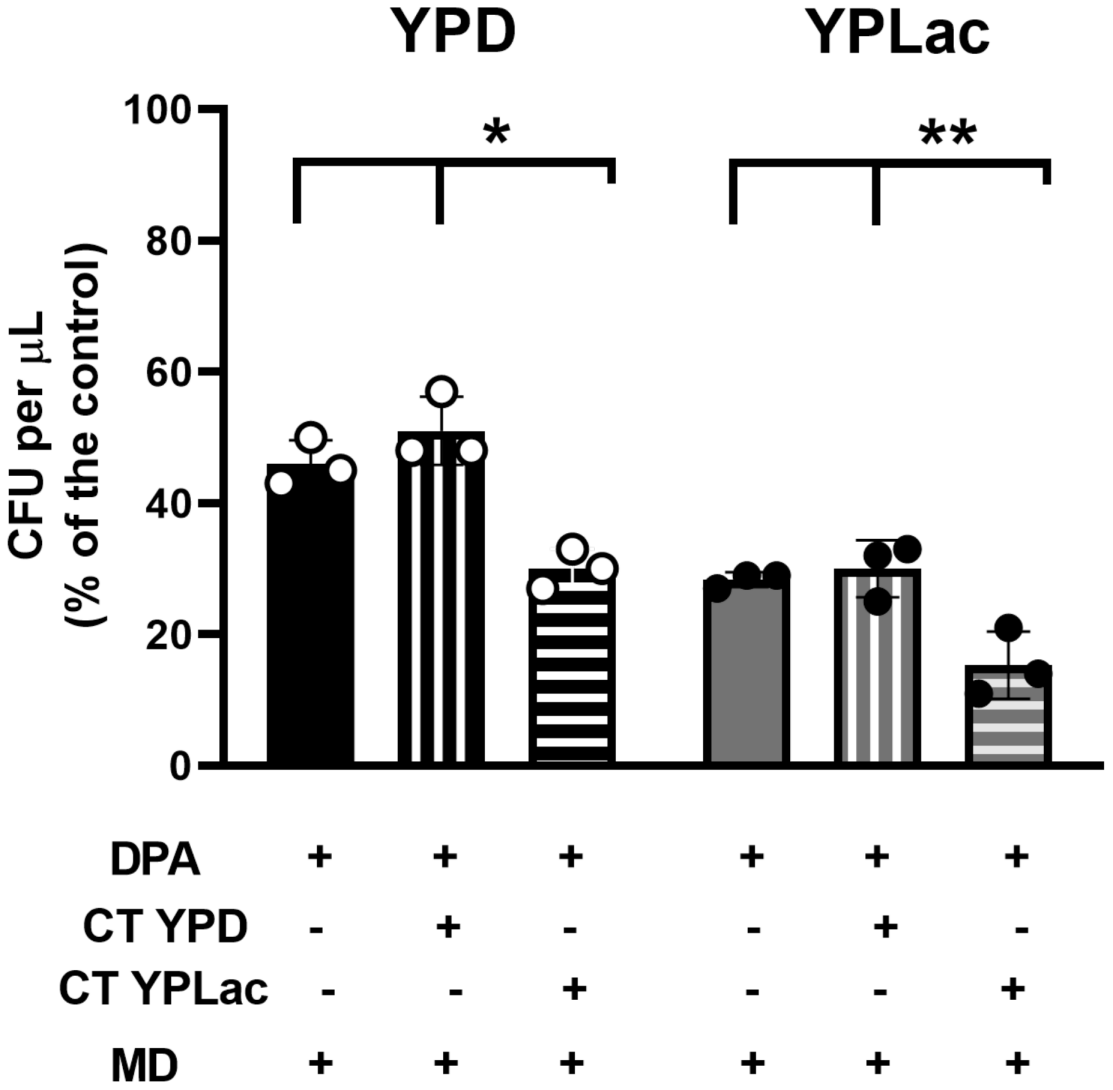
Effect of previously extracted carotenoids on cell viability using a CFU assay. Cells were incubated as in **Figure 1** with 15 µM DPA for 24 h. Then 40 mM menadione was added to all samples, alone (Plain columns) or with 40 µg carotenoid extracts/g cells dry weight from either YPD- (vertical lines) or YPLac-cells (horizontal lines) was added, and cells were further incubated for 2 h at 30°C, shaking at 250 rpm. Next, samples were plated in YPD or YPLac agar at a 10^-5^ dilution and incubated for 3 days at 30°C. These were used to evaluate CFUs YPD-cells Black bars and YPLac Gray bars. Data are shown as mean ± SD (n=3). Where indicated: *(p=0.0051), **(p=0.0122).

After carotenoids interact with free radicals, they may become inactive or even pro-oxidant (Ribeiro et al., 2018; Lucas et al., 2020). To test whether carotenoid inactivation contributed to the increased ROS susceptibility observed in YPLac-cells, we quantified the effects of adding extracted carotenoids to new cells (**Figure 6**). YPLac-cell carotenoid extracts (**Figure 6**, vertical striped bars) or YPD-cell carotenoid extracts (**Figure 6**, horizontal striped bars) were added to cells grown with MD plus DPA in either YPD (**Figure 6**, black bars) or YPLac (**Figure 6**, gray bars). Then, Colony Forming Units (CFUs) were measured. In controls without extracted carotenoids, YPD- (**Figure 6**, black plain bar) and YPLac-grown cells (**Figure 6**, gray plain bar) produced 2.3x10^8^ and 1.2x10^8^ CFUs, respectively (See **Supplementary Figure S3**). When carotenoid extracts from YPD-cells were added back to new cell cultures, a non-significative increase in CFU numbers both in YPD- and YPLac-grown cells was observed (**Figure 6**, vertical striped bars). In contrast, adding YPLac-carotenoid extract resulted in a decrease to about half the number of CFUs both in YPD- and YPLac-cells (**Figure 6**, horizontal-striped bars). These results suggest that in YPLac-cells aerobic metabolism induced high levels of ROS reacted with carotenoids, which were inactivated or even became pro-oxidizing species themselves (Ribeiro et al., 2018; Lucas et al., 2020).

## 4 Discussion

Yeasts thrive on different carbon sources, adjusting their metabolism (Fendt and Sauer, 2010). In *Saccharomyces cerevisiae*, glucose and fructose promote fermentative metabolism while lactate and pyruvate depend on oxidative metabolism (Renvoisé et al., 2014). Fermentative metabolism induces catabolic repression, decreasing TCA and respiratory-chain enzyme expression (Gancedo, 1998; Renvoisé et al., 2014). Although oxidative phosphorylation is an efficient ATP producing pathway, it is not favored by yeast due to its slower rate. In addition, redox reactions may overproduce deleterious ROS.

Catalyzed oxygen reduction is highly exergonic and provides high amounts of free energy to sustain life (Nicholls and Ferguson, 2013). However, it may also react spontaneously to yield highly mobile free radicals known as the Reactive Oxygen Species (ROS). ROS react with organic molecules such as proteins, nucleic acids and lipids evoking dysfunction and death (Jomova et al., 2023). Since the Great Oxygenation Event (GOE), only those organisms that can manage ROS toxicity survived (Rosas-Lemus et al., 2016; Mendez-Romero et al., 2022). ROS production in the cell may be prevented by many mechanisms, including uncoupling of oxidative phosphorylation (Guerrero-Castillo et al., 2011) or hiding, in biofilms (Jarros et al., 2020) or behind an impermeable epithelium (Rosas-Lemus et al., 2016). *Rhodotorula* spp is exceptional in that it possesses most stress-defense systems: it is protected from contaminating agents by an extracellular matrix (Cho et al., 2001), it can associate into biofilms (Jarros et al., 2020), its mitochondrial respiratory chain is highly branched (Castañeda-Tamez et al., 2024), it expresses ROS detoxifying enzymes (Li and Ma, 2021) and in addition, it produces carotenoids that inactivate ROS produced by UV radiation (Garcia-Cortes et al., 2021) of by oxidative stress (Chen et al., 2022).

For the first billion years after life began, anaerobic life flourished. Unicellular prokaryotes and eukaryotes populated the Earth (Lane, 2002). Then, about two and a half billion years ago GOE, where oxygen concentration rose about 10^5^ times, led to the first mass extinction (Lane, 2002). Oxygen reduction releases large amounts of energy during its physiological reduction (Mendez-Romero et al., 2022). However, a special kind of FR, the highly motile, toxic ROS may be produced in spontaneous side reactions (Li et al., 2018; Sies et al., 2022). Once ROS are produced, these are deactivated by enzymes like superoxide dismutase, catalases and the glutathione system (Jamova et al., 2024) or by pigments like chlorophylls, melanin and carotenoids (Stahl and Sies, 2003; Salman et al., 2007; Choi and Lee, 2015; Lucas et al., 2020; Priyadarshini Pradhan et al., 2022; Tamiaki, 2022; Suthar et al., 2023). In humans, ingested carotenoids can protect against cancer and various illnesses, including cardiovascular disorders, cataracts, age-related macular degeneration, osteoporosis, and diabetes (Milani et al., 2017; Shabhir and Nuzhat, 2018; Paul et al., 2023).

The mitochondrial respiratory chain produces FR, mostly in the NADH/ubiquinone oxido-reductase (Complex I), and in the ubiquinone/cytochrome-*c* oxido-reductase (Complex III) (Mazat et al., 2020). To prevent ROS overproduction, unicellular organisms, plants and crustaceans express branched respiratory chains, where a high rate of electron flow does not give FR enough time to spontaneously react with O_2_ (Guerrero-Castillo et al., 2011; Cabrera-Orefice et al., 2014; Castañeda-Tamez et al., 2024). In contrast to mitochondrial oxygen consumption, fermentation does not produce free radicals, and thus most unicellular species decrease expression of mitochondria either in the absence of oxygen or when supplied with fermentative substrates (Malecki et al., 2020; Malina et al., 2021). When oxidative metabolism is needed mitochondria are expressed, increasing the risk of oxidative damage.

Oxidative stress promotes carotenoid production, retention and bioavailability: in *Bacillus pseudofirmus* OF4, carotenoids contribute to resist oxidative stress during growth at high pH (Hicks et al., 2019). Similarly, in *Blakeslea trispora* during submerged fermentation, oxidative stress triggers antioxidant enzyme activity, enhancing carotenoid synthesis (Roukas, 2015). Additionally, aerobic growth conditions in *Enterococcus gilvus* up-regulate carotenoid biosynthesis genes, which results in enhanced survival (Hagi et al., 2014). Furthermore, in *Xanthophyllomyces dendrorhous* higher oxygen supply increases astaxanthin biosynthesis, while oxygen limitation inhibits growth (Wang and Yu, 2009). Lastly, regulation by ROS enhances growth in *Rhodobacter sphaeroides* under autotrophic conditions, resulting in improved cell growth and increased carotenoid levels (Lee et al., 2022). Here, *R. mucilaginosa* did increase carotenoid synthesis when oxidative metabolism was activated. In addition, our results strongly indicate that the role of carotenoids was to deactivate the high amount of ROS produced by mitochondrial activity.

In our hands, *R. mucilaginosa* growth curves were similar to those reported for other *Rhodothorula* species where biomass yield is enhanced in dextrose (Aksu and Eren, 2005; Ferrao and Garg, 2011; Xu et al., 2011; Szotkowski et al., 2019; Byrtusová et al., 2021). In lactate, growth yields were lower (**Figure 1**) while ROS and carotenoid levels increased, indicating that these cells were under oxidative stress (Sakaki et al., 2002; Lee et al., 2022). Carotenoids react with ROS, inactivating them. However, these reactions may modify carotenoids, which can be inactivated and even become pro-oxidant species (Landolfo et al., 2019). Modifications like these are suggested by our TLC experiments, where a carotenoid band exhibited a different running pattern (**Figure 3C**) (Wall, 2005). Again, in contrast to non-substituted carotenoids such a β-carotene and torulene, torularhodin contains a carboxyl, and thus it is likely that its ROS sensitivity is higher (Sli-Gel et al., 1987; Britton, 2008). Indeed, it has been reported that carotenoids with oxygen substituents react to high oxygen and ozone producing enantiomers and other oxidized derivatives with different migration patterns in TLC (Britton, 2008). Enhancing carotenoid production by subjecting cells to stress seems to be common practice (Shi et al., 2020; Eun and Lee, 2024). Our results suggest that care should be exercised when industrially producing carotenoids, due to the possible deterioration of the desired products promoted by the stress condition used to increase their production.

In other yeast species such as *Kluyveromyces marxianus* cultures grown in ethanol oxidative metabolism increases. This results in higher catalase expression increase suggesting cells are under oxidative stress (Koleva et al., 2008). In *Rhodotorula glutinis* increased ROS also stimulates carotenoid synthesis (Sakaki et al., 1999). In *Debaryomyces hansenii*, the expression of alternative components of the mitochondrial respiratory chain is higher when cultured in YPLac than in YPD (Cabrera-Orefice et al., 2014). Our data revealed that oxidative metabolism in cells grown in YPLac induces oxidative stress, leading to increase synthesis of carotenoids (**Figure 3**). The heightened menadione sensitivity of cells grown in YPLac further confirms a state of oxidative stress (**Figures 3**, **4**, **6**). In addition to oxidative stress, the enhancement of carotenoid production may have resulted from higher availability of as pyruvate and acetyl-CoA, which are derived from lactate metabolism (Somashekar and Joseph, 2000; Chaturvedi et al., 2021).

*Rhodotorula* species produce torularhodin, torulene and β-carotene (Perrier et al., 1995; Moliné et al., 2012; Kot et al., 2019; Tang et al., 2019). These were probably present in our extracts as suggested by absorbance spectra and TLC (**Figures 5A–C**) (Park et al., 2007; Cheng and Yang, 2016; Varmira et al., 2016). It has been suggested that carotenoid proportions vary with the carbon source (Lucas et al., 2020) and *R. mucilaginosa* growing on Minimal Medium contains 60-80% torularhodin and 10-20% β-carotene, while torulene can be found in negligible amounts (Moliné et al., 2012). As expected, in *R. mucilaginosa* grown in lactate, carotenoid synthesis increased (**Figure 3**).

## 5 Conclusion

Under oxidative stress *R. mucilaginosa* increases carotenoid production. Inhibiting carotenoid synthesis unmasked a high concentration in YPLac-grown cells (**Figure 4**). This highlights the protective role of carotenoids in *R. mucilaginosa* (**Figures 5**, **6**), which has already been reported by others (Maxwell et al., 1966; Valadon and Mummery, 1966; Moore et al., 1989; Baltschun et al., 1997; Stahl et al., 1998; Boussiba, 2000; Irazusta et al., 2013). Carotenoid-mediated protection was not needed by YPD-grown cells, suggesting that under these conditions few ROS were present. Remarkably, carotenoids in YPLac-grown cells were most likely modified after ROS exposure (Henry et al., 2000), such that their addition decreased survival in YPLac-grown *R. mucilaginosa* cells (**Figure 6**). The exact identity of native and modified carotenoids was not confirmed. To do this, mass spectrometry experiments have to be conducted on the bands resolved by TLC (**Figure 4**).

## Data Availability

The original contributions presented in the study are included in the article/**Supplementary Material**. Further inquiries can be directed to the corresponding authors.

## Supplementary material

Supplementary Figure 1
*Rhodotorula mucilaginosa* growth curves in the presence of the carotenoid synthesis inhibitor diphenylamine (DPA). Media were **(A)** YPD (black) or **(B)** YPLac (gray). Conditions included: control, continuous line, 15 µM DPA (dashed line) and 40 µM DPA (continuous line). Experimental conditions as in **Figure 1**. DPA was added in 40 μL of 95% ethanol from appropriate stock solutions.

Supplementary Figure 2 Representative oxymetry traces. Cells were added to a final concentration of 12.5 mg (ww)/mL. Where indicated additions were: 40 µM DPA and 32 µM carbonyl cyanide 3-chlorophenylhydrazone (CCCP). Added cells were from cultures grown in **(A)** YPD or **(B)** YPLac.

Supplementary Figure 3 Effect of previously extracted carotenoids on cell viability using a CFU assay. The data used to make **Figure 6** are presented to show original CFU numbers in each YPD and YPLac samples. Empty Bars reporting CFUs without any additions are included. All others are as in **Figure 6**: Plain bars, menadione without carotenoids. Striped bars: 40 μg carotenoid extracts/g cells dry weight from either YPD- (vertical lines) or YPLac-cells (horizontal). Cells were incubated for 2 h at 30°C, shaking at 250 rpm. Samples were plated in YPD or YPLac agar at a 10^-5^ dilution and incubated for 3 days at 30°C. These were used to evaluate CFUs. YPD-cells Black bars and YPLac Gray bars.
