# Supplementary figures and images for: In Rhodotorula mucilaginosa, active oxidative metabolism increases carotenoids to inactivate excess reactive oxygen species
Source: Front Fungal Biol. 2024 Sep 6;5:1378590. doi: 10.3389/ffunb.2024.1378590 (PMC11412819; doi:10.3389/ffunb.2024.1378590)

**Fig. S1.**

**
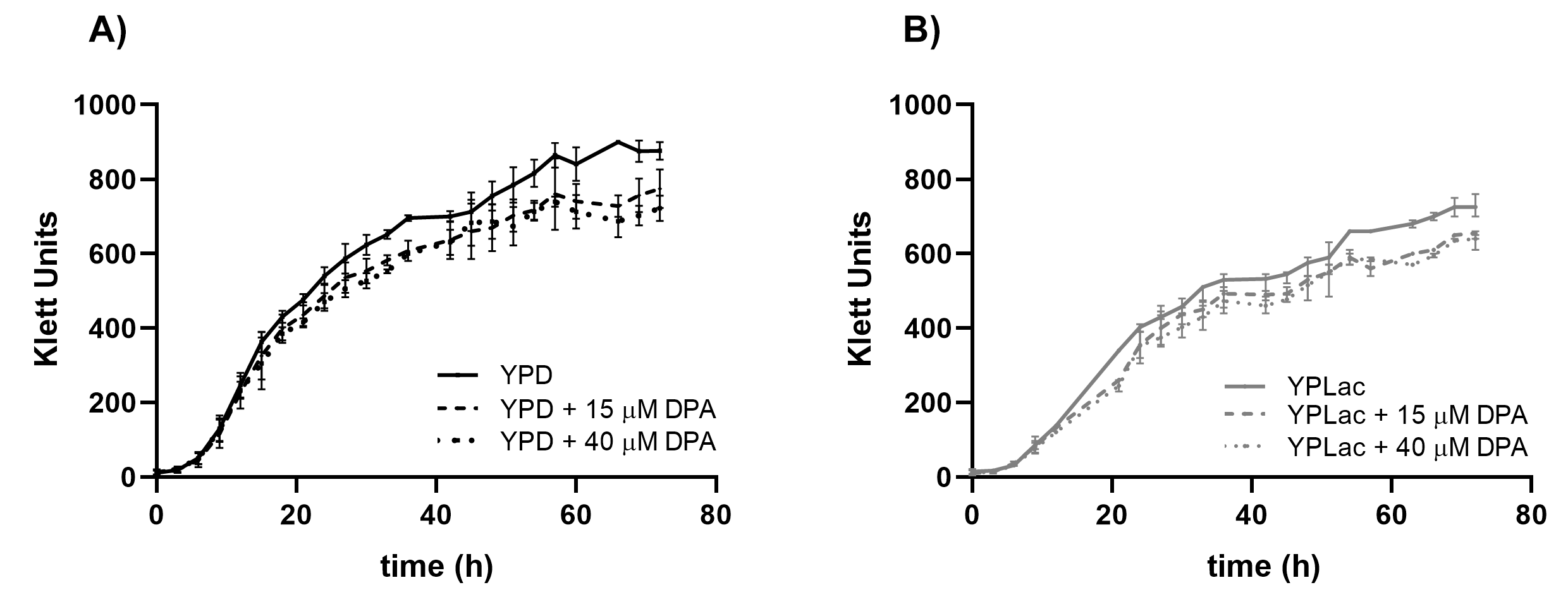
**

**Fig. S2**

**
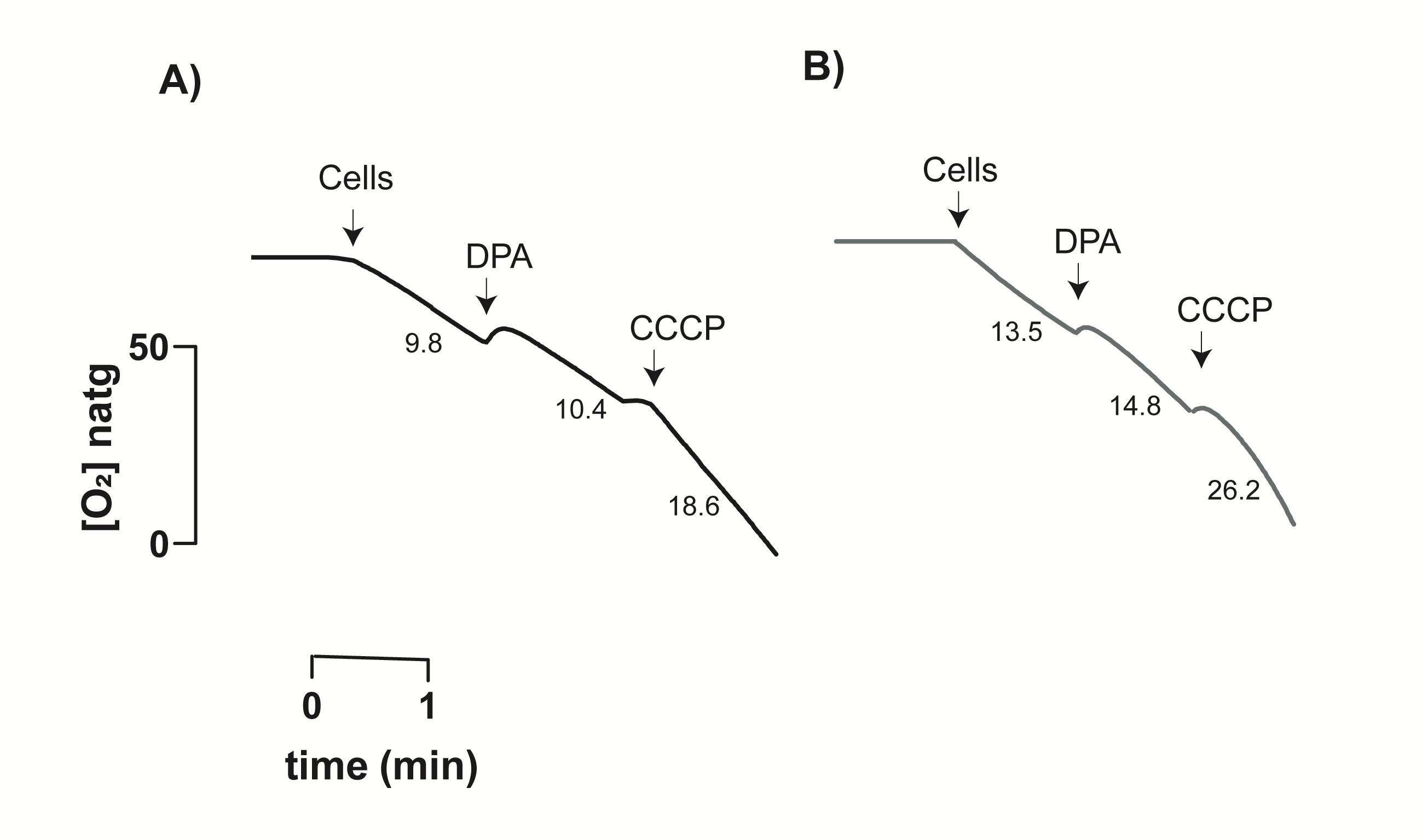
**

**Fig. S3**

**
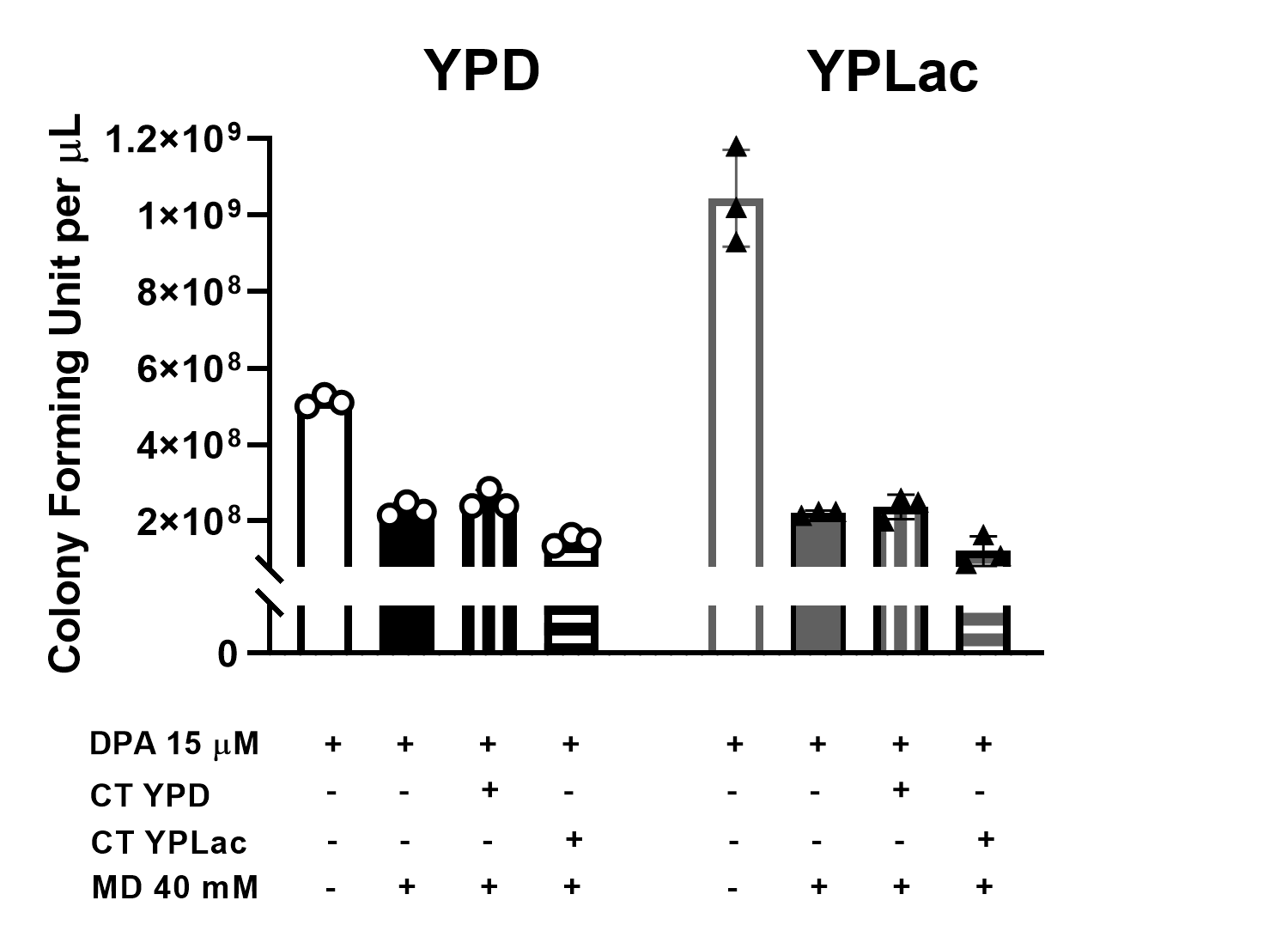
**

Supplement: Supplementary Figure 1 — Rhodotorula mucilaginosa growth curves in the presence of the carotenoid synthesis inhibitor diphenylamine (DPA). Media were (A) YPD (black) or (B) YPLac (gray). Conditions included: control, continuous line, 15 µM DPA (dashed line) and 40 µM DPA (continuous line). Experimental conditions as in Figure 1 . DPA was added in 40 μL of 95% ethanol from appropriate stock solutions. [file DataSheet1.docx]
